# Association of Transthyretin Valine to Isoleucine Variant with Kidney Outcomes in Community-Dwelling Black Adults: The Reasons for Geographic and Racial Differences in Stroke Cohort

**DOI:** 10.34067/KID.0000000935

**Published:** 2025-07-29

**Authors:** Soumya Khanna, Katharine L. Cheung, Pankaj Arora, Marguerite R. Irvin, Leslie A. Lange, Titi Ilori, Mary Cushman, Akhil Pampana, Orlando M. Gutierrez

**Affiliations:** 1 Marnix Heersink School of Medicine, University of Alabama at Birmingham, Birmingham, Alabama; 2 Department of Medicine, Larner College of Medicine at the University of Vermont, Burlington, Vermont; 3 Division of Cardiovascular Disease, Department of Medicine, Marnix Heersink School of Medicine, University of Alabama at Birmingham, Birmingham, Alabama; 4 Department of Epidemiology, School of Public Health, University of Alabama at Birmingham, Birmingham, Alabama; 5 Department of Epidemiology, Colorado School of Public Health, Denver, Colorado; 6 Division of Nephrology, Boston University School of Medicine, Boston, Massachusetts; 7 Division of Nephrology, Department of Medicine, Marnix Heersink School of Medicine, University of Alabama at Birmingham, Birmingham, Alabama

**Keywords:** CKD, epidemiology and outcomes, health equity, diversity, and inclusion, human genetics, minority health and disparities

Hereditary transthyretin amyloidosis (ATTRv) is an autosomal dominant condition in which mutations of the transthyretin (*TTR*) gene disrupt *TTR* tetramer interactions and decrease protein folding stability, resulting in monomer aggregation and amyloid fibril formation.^1^ Depending on the specific *TTR* mutation, amyloid deposits can occur in a variety of tissues including the myocardium, nervous system, and kidneys.^1,2^ Over 150 variants in the *TTR* gene have been identified.^3^ The single-amino acid mutation from valine to isoleucine (Val122Ile) is the most common variant in Black Americans.^4^ This specific mutation leads to concentric left ventricular hypertrophy and is associated with higher risk of incident heart failure in Black Americans.^5^ A previous Reasons for Geographic and Racial Differences in Stroke (REGARDS) study demonstrated that Black American carriers of the *TTR* Val122Ile variant were at significantly higher risk of heart failure, heart failure mortality, cardiovascular mortality, and all-cause mortality.^2^

ATTRv can also lead to extracellular deposition of amyloid proteins in kidney tissues resulting in proteinuria and CKD.^3,6^ Most studies describing the impact of ATTRv on the kidney focused on the *TTR* Val30Met mutation which is the most common mutation of ATTRv worldwide and is found predominantly in Portuguese populations.^3^ Kidney involvement in patients with *TTR* Val122Ile mutations has been studied in less detail. A recent study of French adults with known ATTRv showed that 30% had evidence of CKD. Moreover, this study showed that carriage of the Val122Ile mutation was associated with a substantially high risk of CKD in fully adjusted models (odds ratio, 61; 95% confidence interval [CI], 6.2 to 8334).^6^ Whether a similar association is observed in other populations is unclear. Therefore, in 8669 Black participants of the REGARDS study with complete data on *TTR* genotyping and kidney function at the baseline visit, we examined the association of the *TTR* Val122Ile (rs76992529) genotype with (1) eGFR and urine albumin-to-creatinine ratios (ACR) at baseline and (2) risk of developing incident ESKD over time. eGFR was determined using the CKD-Epidemiology Collaboration 2021 equation using creatinine and cystatin C without the race variable.^7^ Data on incident ESKD were derived from linkage to the United States Renal Data System through June 2018.

A total of 265 participants were carriers of the *TTR* Val122Ile variant and 8404 were noncarriers. There were no significant differences in baseline characteristics between the two groups, including baseline eGFR and ACR (Table 1). In linear regression analyses adjusted for age, sex, and genetic ancestry, there were no significant associations of *TTR* Val122Ile carriage with eGFR (*β* estimate, 0.77; SEM, 1.38; *P* = 0.56) or ACR (*β* estimate, −0.06; SEM, 0.09; *P* = 0.50; Table 2). The results did not differ after further adjustment for body mass index, education, income level, smoking status, systolic BP, lipids, left ventricular hypertrophy, diabetes, coronary heart disease, stroke, and ACR (in models with eGFR as the dependent variable) or eGFR (in models with ACR as the dependent variable).

**Table 1:** Baseline characteristics of study participants by transthyretin valine to isoleucine variant status

Characteristics	*TTR* Val122Ile Variant Status	
	Noncarriers (*n*=8404)	Carriers (*n*=265)
**Demographics**		
Age, yr; mean (SD)	63 (9)	63 (9)
Male sex, %	39	37
Region of residence, %		
*Stroke belt*	34	33
*Stroke buckle*	17	18
*Other*	49	49
**Social factors**		
Income <$20,000, %	25	25
<High school graduate, %	18	19
**Risk factors**		
Current smoker, %	82	79
No exercise, %	36	34
BMI, mean (SD)	31 (7)	30 (6)
BP, mm Hg, mean (SD)		
*Systolic*	131 (17)	130 (17)
*Diastolic*	78 (10)	78 (10)
eGFR, ml/min per 1.73 m^2^, mean (SD)	83 (23)	84 (23)
ACR, mg/g, median (IQR)	8.0 (4.7–20.77)	7.6 (4.9–18.6)
**Comorbidities, %**		
Coronary heart disease	14	15
Diabetes status	29	29
Hypertension	65	66
Stroke	7	8

ACR, albumin-to-creatinine ratio; BMI, body mass index; IQR, interquartile range; *TTR*, transthyretin; Val122Ile, valine to isoleucine.

**Table 2:** Cross-sectional association of carriers versus noncarriers with eGFR and urine albumin to creatinine ratio at baseline

Variable	*β* estimate (SEM)	*P* Value
**eGFR**		
Crude	1.22 (1.48)	0.41
Model 1	0.77 (1.34)	0.56
Model 2	0.71 (1.33)	0.59
Model 3	0.24 (1.27)	0.85
**ACR**		
Crude	−0.07 (0.09)	0.43
Model 1	−0.06 (0.09)	0.50
Model 2	−0.05 (0.09)	0.59
Model 3	−0.05 (0.09)	0.57

Model 1 adjusts for age, sex, and the first ten principal components of ancestry.

Model 2 adjusts for variables in model 1+body mass index, education (high school graduate or above versus non–high school graduate) income level (<$35 000 versus ≥$35 000), smoking status (current, past, or never smoker), systolic BP, lipid parameters, and a history of left ventricular hypertrophy (by electrocardiogram criteria), diabetes, coronary heart disease, and stroke.

Model 3 adjusts for eGFR in models where albumin-to-creatinine ratio is the dependent variable and albumin-to-creatinine ratio in models where eGFR is the dependent variable. ACR, albumin-to-creatinine ratio.

After a mean 8±3 years of follow-up, a total of 400 participants developed ESKD (ten in carriers of the *TTR* Val122Ile variant and 390 in noncarriers). The incidence of ESKD per 1000 person-years of follow-up was 357 (95% CI, 191 to 663) in carriers of the *TTR* Val122Ile variant and 431 (95% CI, 391 to 476) in noncarriers. There was no significant association of *TTR* Val122Ile with incident ESKD in models adjusted for age, sex, and genetic ancestry (hazard ratio, 0.81; 95% CI, 0.43 to 1.52) or in fully adjusted analyses (hazard ratio, 0.59; 95% CI, 0.25 to 1.44). In addition, there was no interaction between the *TTR* Val122Ile variant with *APOL1* nephropathy risk variants. Thus, in Black participants of the REGARDS study, carriage of the *TTR* Val122Ile variant was not associated with prevalent CKD or with incident ESKD.

These results should be interpreted in the context of several limitations. First, the number of individuals with the *TTR* Val122Ile variant was relatively small, which may have limited statistical power to detect more modest associations. In addition, the participants' age was older at baseline. This might cause survivor bias if carriers of the *TTR* Val122Ile variant tended to die at younger ages and could not be enrolled in the REGARDS study. Finally, we did not have data on local ancestry specific genome-wide association studies, which might help identify local haplotypes that may explain differences in the association of this variant with kidney disease in this study as compared with previous studies of predominantly White individuals.

In summary, although previous studies in mostly White populations showed strong associations of the *TTR* Val122Ile variant with prevalent CKD, we did not find an association of carriage of this variant with kidney disease in older, community-dwelling Black adults. These results suggest that the implication of *TTR* Val122Ile variants on kidney health may differ by genetic ancestry.

## Supplementary Material

SUPPLEMENTARY MATERIAL

## Data Availability

All data are included in the manuscript and/or supporting information.

## Declarative Statements

This study includes clinical experimentation and received Institutional Review Board or Ethics Committee approval. The need to obtain informed patient consent was waived. This study includes clinical experimentation and complies with the Declaration of Helsinki.
