# Supplementary material for: Association of Transthyretin Valine to Isoleucine Variant with Kidney Outcomes in Community-Dwelling Black Adults: The Reasons for Geographic and Racial Differences in Stroke Cohort
Source: Kidney360. 2025 Jul 29;6(12):2248–50. doi: 10.34067/KID.0000000935 (PMC12708367; doi:10.34067/KID.0000000935)
Supplement: SUPPLEMENTARY MATERIAL [file kidney360-6-2248-s001.pdf]

ASN
American Society of Nephrology
JASN
Journal of the American Society of Nephrology
KIDNEY360
Accessing Our World From Every Angle
CJASN
Clinical Journal of the American Society of Nephrology

ASN Journal Disclosure Form

As per ASN journal policy, I have disclosed any financial relationships or commitments I have held in the past 36 months as included below. I have listed my Current Employer below to indicate there is a relationship requiring disclosure. If no relationship exists, my Current Employer is not listed.

P. Arora reports the following:
Employer: University of Alabama at Birmingham; Consultancy: Bristol-Myers Squibb; and Research Funding: Dr. Pankaj Arora is supported by the National Heart, Lung, and Blood Institute of the National Institutes of Health (NIH) awards (R01HL160982, R01HL163852, and R01HL163081).

I understand that the information above will be published within the journal article, if accepted, and that failure to comply and/or to accurately and completely report the potential financial conflicts of interest could lead to the following: 1) Prior to publication, article rejection, or 2) Post-publication, sanctions ranging from, but not limited to, issuing a correction, reporting the inaccurate information to the authors' institution, banning authors from submitting work to ASN journals for varying lengths of time, and/or retraction of the published work.

Name: Pankaj Arora
Manuscript ID: K360-2025-000085R1
Manuscript Title: Association of Transthyretin Val122Ile Variant with kidney outcomes in community-dwelling Black adults: the REGARDS cohort
Date of Completion: July 7, 2025
Disclosure Updated Date: July 7, 2025

ASN American Society of Nephrology
JASN Journal of the American Society of Nephrology
KIDNEY360 Accessing Our World From Every Angle
CJASN Clinical Journal of the American Society of Nephrology

ASN Journal Disclosure Form

As per ASN journal policy, I have disclosed any financial relationships or commitments I have held in the past 36 months as included below. I have listed my Current Employer below to indicate there is a relationship requiring disclosure. If no relationship exists, my Current Employer is not listed.

K. Cheung reports the following:
Employer: UNIVERSITY OF VERMONT; and Advisory or Leadership Role: Director of Center on Aging at University of Vermont (paid).

I understand that the information above will be published within the journal article, if accepted, and that failure to comply and/or to accurately and completely report the potential financial conflicts of interest could lead to the following: 1) Prior to publication, article rejection, or 2) Post-publication, sanctions ranging from, but not limited to, issuing a correction, reporting the inaccurate information to the authors' institution, banning authors from submitting work to ASN journals for varying lengths of time, and/or retraction of the published work.

Name: Katharine L. Cheung
Manuscript ID: K360-2025-000085R1
Manuscript Title: Association of Transthyretin Val122Ile Variant with kidney outcomes in community-dwelling Black adults: the REGARDS cohort
Date of Completion: May 23, 2025
Disclosure Updated Date: May 23, 2025

ASN American Society of Nephrology JASN Journal of the American Society of Nephrology KIDNEY360 Accessing Our World From Every Angle CJASN Clinical Journal of the American Society of Nephrology

ASN Journal Disclosure Form

As per ASN journal policy, I have disclosed any financial relationships or commitments I have held in the past 36 months as included below. I have listed my Current Employer below to indicate there is a relationship requiring disclosure. If no relationship exists, my Current Employer is not listed.

M. Cushman reports the following:
Employer: University of Vermont; and Advisory or Leadership Role: -Hemostasis and Thrombosis Research Society - board member. unpaid; -International Society on Thrombosis and Haemostasis ; board member, unpaid. For both, travel cost is provided for meetings.

I understand that the information above will be published within the journal article, if accepted, and that failure to comply and/or to accurately and completely report the potential financial conflicts of interest could lead to the following: 1) Prior to publication, article rejection, or 2) Post-publication, sanctions ranging from, but not limited to, issuing a correction, reporting the inaccurate information to the authors' institution, banning authors from submitting work to ASN journals for varying lengths of time, and/or retraction of the published work.

Name: Mary Cushman
Manuscript ID: K360-2025-000085R1
Manuscript Title: Association of Transthyretin Val122Ile Variant with kidney outcomes in community-dwelling Black adults: the REGARDS cohort
Date of Completion: July 3, 2025
Disclosure Updated Date: July 3, 2025

ASN
American Society of Nephrology
JASN
Journal of the American Society of Nephrology
KIDNEY360
Accessing Our World From Every Angle
CJASN
Clinical Journal of the American Society of Nephrology

ASN Journal Disclosure Form

As per ASN journal policy, I have disclosed any financial relationships or commitments I have held in the past 36 months as included below. I have listed my Current Employer below to indicate there is a relationship requiring disclosure. If no relationship exists, my Current Employer is not listed.

O. Gutierrez reports the following:
Employer: UAB School of Medicine; and Honoraria: Akebia; Amgen; Ardelyx.

I understand that the information above will be published within the journal article, if accepted, and that failure to comply and/or to accurately and completely report the potential financial conflicts of interest could lead to the following: 1) Prior to publication, article rejection, or 2) Post-publication, sanctions ranging from, but not limited to, issuing a correction, reporting the inaccurate information to the authors' institution, banning authors from submitting work to ASN journals for varying lengths of time, and/or retraction of the published work.

Name: Orlando M. Gutierrez
Manuscript ID: K360-2025-000085R1
Manuscript Title: Association of Transthyretin Val122Ile Variant with kidney outcomes in community-dwelling Black adults: the REGARDS cohort
Date of Completion: May 25, 2025
Disclosure Updated Date: February 25, 2025

ASN
American Society of Nephrology
JASN
Journal of the American Society of Nephrology
KIDNEY360
Accessing Our World From Every Angle
CJASN
Clinical Journal of the American Society of Nephrology

ASN Journal Disclosure Form

As per ASN journal policy, I have disclosed any financial relationships or commitments I have held in the past 36 months as included below. I have listed my Current Employer below to indicate there is a relationship requiring disclosure. If no relationship exists, my Current Employer is not listed.

T. Ilori reports the following:
Employer: Boston Universiy School of Medicine, Boston Medical Center; and Research Funding: Vertex Pharmaceuticals; NIDDK - K23DK119542, 1R03DK140290-01.

I understand that the information above will be published within the journal article, if accepted, and that failure to comply and/or to accurately and completely report the potential financial conflicts of interest could lead to the following: 1) Prior to publication, article rejection, or 2) Post-publication, sanctions ranging from, but not limited to, issuing a correction, reporting the inaccurate information to the authors' institution, banning authors from submitting work to ASN journals for varying lengths of time, and/or retraction of the published work.

Name: Titilayo O. Ilori
Manuscript ID: K360-2025-000085R1
Manuscript Title: Association of Transthyretin Val122Ile Variant with kidney outcomes in community-dwelling Black adults: the REGARDS cohort
Date of Completion: May 23, 2025
Disclosure Updated Date: May 23, 2025

ASN American Society of Nephrology
JASN Journal of the American Society of Nephrology
KIDNEY360 Accessing Our World From Every Angle
CJASN Clinical Journal of the American Society of Nephrology

ASN Journal Disclosure Form

As per ASN journal policy, I have disclosed any financial relationships or commitments I have held in the past 36 months as included below. I have listed my Current Employer below to indicate there is a relationship requiring disclosure. If no relationship exists, my Current Employer is not listed.

M. Irvin reports the following:
Employer: UAB

I understand that the information above will be published within the journal article, if accepted, and that failure to comply and/or to accurately and completely report the potential financial conflicts of interest could lead to the following: 1) Prior to publication, article rejection, or 2) Post-publication, sanctions ranging from, but not limited to, issuing a correction, reporting the inaccurate information to the authors' institution, banning authors from submitting work to ASN journals for varying lengths of time, and/or retraction of the published work.

Name: Marguerite Irvin
Manuscript ID: K360-2025-000085R1
Manuscript Title: Association of Transthyretin Val122Ile Variant with kidney outcomes in community-dwelling Black adults: the REGARDS cohort
Date of Completion: July 17, 2025
Disclosure Updated Date: July 17, 2025

ASN
American Society of Nephrology
JASN
Journal of the American Society of Nephrology
KIDNEY360
Accessing Our World From Every Angle
CJASN
Clinical Journal of the American Society of Nephrology

ASN Journal Disclosure Form

As per ASN journal policy, I have disclosed any financial relationships or commitments I have held in the past 36 months as included below. I have listed my Current Employer below to indicate there is a relationship requiring disclosure. If no relationship exists, my Current Employer is not listed.

S. Khanna has nothing to disclose.

I understand that the information above will be published within the journal article, if accepted, and that failure to comply and/or to accurately and completely report the potential financial conflicts of interest could lead to the following: 1) Prior to publication, article rejection, or 2) Post-publication, sanctions ranging from, but not limited to, issuing a correction, reporting the inaccurate information to the authors' institution, banning authors from submitting work to ASN journals for varying lengths of time, and/or retraction of the published work.

Name: Soumya Khanna
Manuscript ID: K360-2025-000085R1
Manuscript Title: Association of Transthyretin Val122Ile Variant with kidney outcomes in community-dwelling Black adults: the REGARDS cohort
Date of Completion: May 23, 2025
Disclosure Updated Date: May 23, 2025

ASN American Society of Nephrology
JASN Journal of the American Society of Nephrology
KIDNEY360 Accessing Our World From Every Angle
CJASN Clinical Journal of the American Society of Nephrology

ASN Journal Disclosure Form

As per ASN journal policy, I have disclosed any financial relationships or commitments I have held in the past 36 months as included below. I have listed my Current Employer below to indicate there is a relationship requiring disclosure. If no relationship exists, my Current Employer is not listed.

L. Lange reports the following:
Employer: University of Colorado, Anschutz Medical Campus

I understand that the information above will be published within the journal article, if accepted, and that failure to comply and/or to accurately and completely report the potential financial conflicts of interest could lead to the following: 1) Prior to publication, article rejection, or 2) Post-publication, sanctions ranging from, but not limited to, issuing a correction, reporting the inaccurate information to the authors' institution, banning authors from submitting work to ASN journals for varying lengths of time, and/or retraction of the published work.

Name: Leslie A. Lange
Manuscript ID: K360-2025-000085R1
Manuscript Title: Association of Transthyretin Val122Ile Variant with kidney outcomes in community-dwelling Black adults: the REGARDS cohort
Date of Completion: July 10, 2025
Disclosure Updated Date: July 10, 2025

ASN American Society of Nephrology | JASN Journal of the American Society of Nephrology | KIDNEY360 Accessing Our World From Every Angle | CJASN Clinical Journal of the American Society of Nephrology

ASN Journal Disclosure Form

As per ASN journal policy, I have disclosed any financial relationships or commitments I have held in the past 36 months as included below. I have listed my Current Employer below to indicate there is a relationship requiring disclosure. If no relationship exists, my Current Employer is not listed.

A. Pampana reports the following:
Employer: UAB

I understand that the information above will be published within the journal article, if accepted, and that failure to comply and/or to accurately and completely report the potential financial conflicts of interest could lead to the following: 1) Prior to publication, article rejection, or 2) Post-publication, sanctions ranging from, but not limited to, issuing a correction, reporting the inaccurate information to the authors' institution, banning authors from submitting work to ASN journals for varying lengths of time, and/or retraction of the published work.

Name: Akhil Pampana
Manuscript ID: K360-2025-000085R1
Manuscript Title: Association of Transthyretin Val122Ile Variant with kidney outcomes in community-dwelling Black adults: the REGARDS cohort
Date of Completion: May 19, 2025
Disclosure Updated Date: May 19, 2025
